# Assessing the risk factors for myocardial infarction in diet-induced prediabetes: myocardial tissue changes

**DOI:** 10.1186/s12872-022-02758-8

**Published:** 2022-08-02

**Authors:** Nompumelelo Gumede, Phikelelani Ngubane, Andile Khathi

**Affiliations:** 1 Department of Human Physiology, School of Laboratory Medicine and Medical Sciences, College of Health Sciences, University of KwaZulu-Natal, Durban, X54001 South Africa; 2 Department of Human Physiology, School of Laboratory Medicine and Medical Sciences, College of Health Sciences, University of KwaZulu-Natal, Room E2 401, Westville, South Africa

**Keywords:** Polyol pathway, NADH oxidase, Oxidative stress, Myocardial injury, Antioxidant, Prediabetes

## Abstract

**Background:**

Hyperglycaemia is known to result in oxidative stress tissue injury and dysfunction. Interestingly, studies have reported hepatic and renal oxidative stress injury during prediabetes; however, any injury to the myocardium during prediabetes has not been investigated. Hence this study aims to assess changes in the myocardial tissue in an HFHC diet-induced model of prediabetes.

**Methods:**

Male Sprague Dawley rats were randomly grouped into non-prediabetes and prediabetes (n = 6 in each group) and consumed a standard rat chow or fed a high-fat-high-carbohydrate diet respectively for a 20-week prediabetes induction period. Post induction, prediabetes was confirmed using the ADA criteria. Aldose reductase, NADH oxidase 1, superoxide dismutase, glutathione peroxide, cardiac troponins were analysed in cardiac tissue homogenate using specific ELISA kits. Lipid peroxidation was estimated by determining the concentration of malondialdehyde in the heart tissue homogenate according to the previously described protocol. Myocardial tissue sections were stained with H&E stain and analysed using Leica microsystem. All data were expressed as means ± SEM. Statistical comparisons were performed with Graph Pad instat Software using the Student's two-sided t-test. Pearson correlation coefficient was calculated to assess the association. Value of *p* < 0.05 was considered statistically significant.

**Results:**

The prediabetes group showed a markedly high oxidative stress as indicated by significantly increased NADH oxidase 1 and malondialdehyde while superoxide dismutase and glutathione peroxide were decreased compared to non-prediabetes group. There was no statistical difference between cardiac troponin I and T in the non-prediabetes and prediabetes groups. Cardiac troponins had a weak positive association with glycated haemoglobin.

**Conclusion:**

The findings of this study demonstrate that prediabetes is associated with myocardial injury through oxidative stress. Future studies are to investigate cardiac contractile function and include more cardiac biomarkers.

**Supplementary Information:**

The online version contains supplementary material available at 10.1186/s12872-022-02758-8.

## Background

Prediabetes is a state of intermediate hyperglycaemia with glucose levels above normal but below the diabetic threshold [1]. Prediabetes is characterized by impaired fasting glucose (IFG), impaired glucose tolerance (IGT) and glycated haemoglobin (Hb1Ac) [2]. These parameters of prediabetes are risks for progressing to type 2 diabetes mellitus and its complications(T2DM) [3, 4]. Approximately 25% of individuals with prediabetes will progress to T2DM and 70% of those individuals will develop overt diabetes complications within their lifetime [5, 6]. Cardiovascular disease (CVD) is the leading cause of mortality and morbidity in T2DM worldwide and myocardial infarction (MI) contributes significantly to CVD mortality [7–9]. In T2DM reactive oxygen species (ROS) generation within the cardiomyocytes exceeds antioxidant defence leading to oxidative damage [10, 11]. The polyol pathway depletes nicotinamide adenine dinucleotide phosphate (NADPH), which is essential for generating glutathione while producing NADH, a substrate for NADH oxidase [12]. The expression of aldose reductase (AR) and sorbitol dehydrogenase (SDH) is increased in the diabetic heart [13]. Studies have reported that AR expression accelerates atherosclerosis in diabetic mice [14]. Atherosclerosis reduces myocardial blood flow and subsequently leads to myocardial infarction (MI) [15]. Furthermore, overexpression of AR in cardiomyocytes leads to a greater infarct area [16, 17].

Alternatively, hyperglycaemia in T2DM increases NADH oxidase1 [18]. NADH oxidase1 is one enzyme that produces excessive ROS within a diabetic heart [18]. Studies show that overexpression of NADH oxidase 1 impairs endothelial vaso-relaxation in animal models of T2DM and may lead to MI [19]. Cardiac troponins (cTnI) and (cTnT) are released as an indication of myocardial damage due to myocardial ischemic injury [20]. Loss of viable myocardium is histological evidence of MI [21]. Elevation of NADH oxidase 1 is reported to play a role in endotoxin-induced cardiomyocyte apoptosis [22].

Studies have demonstrated that T2DM related complications begin during prediabetes [23, 24]. Studies in our laboratory using the high-fat-high-carbohydrate (HFHC) diet-induced model of prediabetes have demonstrated that kidney and brain injury occurs during prediabetes [25, 26]. However, any injury to the myocardium during prediabetes have not been investigated. Hence this study aims to assess changes in myocardial tissue in an HFHC diet-induced model of prediabetes.

## Materials and methods

### Aim

This study aimed to assess myocardial tissue injury in diet-induced prediabetes.

### Animals

Male Sprague–Dawley (150–180 g) rats were obtained from the Biomedical Research Unit (BRU), University of KwaZulu Natal (UKZN). The animals were kept under standard experimental conditions at room temperature (225 ± 2 °C), humidity (55 ± 5%), and 12 h day:12 h night cycle. The animals consumed a standard rat chow (Meadow Feeds, South Africa) and water ad libitum for two weeks to acclimatize before being exposed to an experimental diet (high-fat high carbohydrate). The high-fat high carbohydrate (HFHC) is composed of carbohydrates (55%kcal/g), fats (30%kcal/g), and proteins (15%kcal/g) as previously described [27]. All experimental procedures were conducted in line with ARRIVE guidelines and according to the ethics and animal care guidelines of the Animal Research Ethics Committee (AREC) of UKZN, Durban, South Africa. The study was approved by the UKZN AREC (Ethics No: AREC024/018D).

### Experimental design

After two weeks of acclimatization, the animals were grouped into a non-prediabetic (n = 6) control group and prediabetic group (n = 6). The non-prediabetic group was fed standard rat chow and water ad libitum*.* The prediabetes group was fed an HFHC diet and water supplemented with fructose (15%) for 20 weeks to induce prediabetes. After 20 weeks, prediabetes was confirmed using the American Diabetes Association (ADA) criteria for diagnosis of prediabetes. Animals with fasting blood glucose (FBG) concentrations of 5.6–7.1 mmol/L, oral glucose tolerance test (OGTT) 2-h glucose concentration of 7.1–11.1 mmol/L and glycated haemoglobin (Hb1Ac) concentration of 5.7–6.4% were considered prediabetic. FBG was determined using the tail-prick method and measured using a One-Touch select glucometer (Lifescan, Malta, United Kingdom). OGTT was conducted as per laboratory established protocol [27–31]. Briefly, after 12 h fasting period, FBG was measured (time, 0 min) in all the animals. Glucose (0.86 g/kg, p.o.) was loaded into the animals via oral gavage (18-gauge gavage needle, 38 mm long curved with 21/4 mm ball end). Glucose concentrations were measured at 30-, 60-, and 120-min following glucose loading.

### Tissue harvesting

The animals were placed in a gas chamber (BRU, UKZN, South Africa) and anesthetized with 100 mg/kg of Isoform (Safeline Pharmaceuticals Ltd, Roodeport, South Africa) for 3 min to collect blood samples. Blood samples were collected by cardiac puncture into precooled heparinized containers in an unconscious state. The blood samples were centrifuged (Eppendorf centrifuge 5403, Germany) at 4 °C , 503 g for 15 min to collect plasma. The plasma was stored at – 80 °C in a Bio Ultra freezer (Snijders Scientific, Tilburg, Holland). The hearts of all the animals were excised, cut in half, rinsed with cold standard saline solution, weighed, and snapped frozen in liquid nitrogen before storage in Bio Ultra freezer at – 80 $^\circ{\rm C}$ for biochemical analysis and in formalin for histological analysis [30, 32].

### Biochemical analysis

#### Oxidative stress

##### Aldose reductase, NADH oxidase 1 and Malondialdehyde (MDA)

Aldose reductase and NADH oxidase 1 activity was measured in the heart tissue homogenate using their respective sandwich ELISA according to the manufacturer’s protocol (Fine Biotech Co., Ltd., Wuhan, China). Lipid peroxidation was estimated by determining the concentration of MDA in the heart tissue homogenate according to a previously described protocol [30, 33].

##### SOD and GPx

The antioxidant status of the heart homogenates was determined by using a specific ELISA kit to analyse the concentration of superoxide dismutase (SOD) and glutathione peroxidase (GPx) according to the instructions of the manufacturer (Elabscience Biotechnology Co., Ltd., Houston, TX, USA).

#### Cardiac injury

##### cTnT and cTnI

Cardiac troponins (cTnT and cTnI) were measured from heart tissue homogenate using specific rat sandwich ELISA according to the protocol from the manufacturer (Elabscience Biotechnology Co., Ltd., Houston, TX, USA).

### Heart tissue histology

Heart tissues were fixed in formalin overnight, paraffin-embedded and processed for sectioning. 0.5 μM sections (Robert-Bosch-Straße, Walldorf, Baden-Württemberg, Germany) were made and stained with haematoxylin and eosin (H&E) to analyse the cardiomyocyte size and the arrangement of cardiomyocyte myofibers and apoptotic cells using Leica microsystems for analysis Leica Scanner, SCN400 and Slide Path Gateway LAN software for analysis (Leica Microsystems CMS, Wetzlar, Germany).

### Statistical analysis

All data were expressed as means ± SEM. Statistical comparisons were performed with Graph Pad instat Software (version 5.00, GraphPad Software, Inc., San Diego, California, USA) using the Student's unpaired two-sided t-test. Pearson correlation coefficient was calculated to find the association between HbA1c and cardiac troponins. A value of *p* < 0.05 was considered statistically significant.

## Results

### Body weight and heart weight

**Fig. 1:**
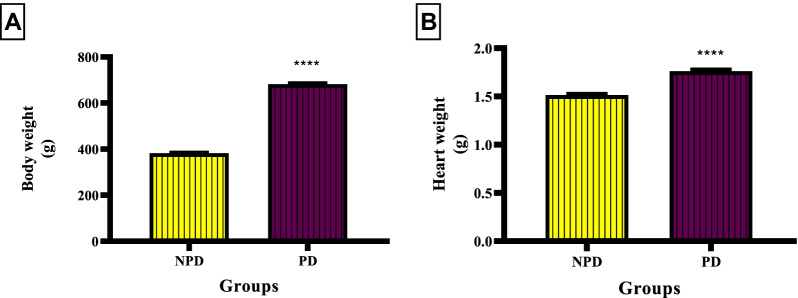
Body weight and heart weight between the NPD and PD group. Values are presented as mean ± SEM. (n = 6 in each group).** A** **** indicates p < 0.0001.** B** **** indicates p < 0.0001. NPD NPD, non-prediabetes; PD, prediabetes; g, gram

**Fig. 2:**
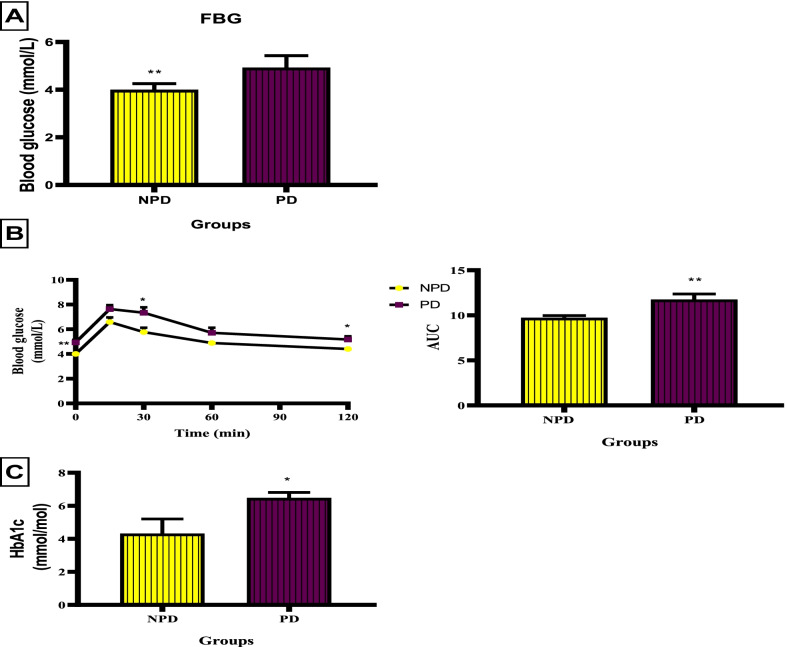
FBG, OGTT and AUC and HbA1c between the NPD and PD group. Values are presented as mean ± SEM (n = 6 in each group). A * Indicates *p* = 0.0020. **B** * Indicates *p* = 0.0215, *p* = 0.0386. ** Indicates *p* = 0.0020, 0.0095. C* Indicates *p* = 0.043. NPD, non-prediabetes; PD, prediabetes; FBG, fasting blood glucose; OGTT, oral glucose tolerance; AUC, area under the curve; HbA1c, glycated haemoglobin

Body weight and heart weight was measured at the end of prediabetes induction period between the NPD and PD groups. Figure 1B shows a significant increase (*p* < 0.0001) in body weight in the PD (681.7 ± 6.22 g) compared to the NPD (382.5 ± 2.54 g) group. Figure 2B shows a significant increase (*p* < 0.0001) in heart weight in the PD group (1.76 ± 0.02 g) compared to the NPD group (1.52 ± 0.01 g).

### ADA prediabetes diagnosis parameters

FBG, OGTT and HbA1c concentration was measured at the end of prediabetes induction between the NPD and PD groups. Figure 2A shows that there was a significant increase (*p* = 0.0020) in FBG in the PD (4.93 ± 0.20 mmol/L) group compared to the NPD group (4.00 ± 0.103 mmol/L). Figure 2B shows that glucose concentration started significantly high in the PD group (*p* = 0.0020) compared to the NPD group at time 0. Glucose concentration remained significantly higher in the PD compared to the NPD as depicted at times 30 min and 120 min with *p* = 0.0215 and *p* = 0.0386, respectively. The AUC graph in Fig. 2B shows a significant increase (*p* = 0.0095) in glucose concentration in the PD group compared to NPD. Figure 2C shows that there was a significant increase (*p* = 0.043) in HbA1c concentration in the PD group (6.49 ± 0.32 mmol/mol) compared to the NPD group (4.32 ± 0.89 mmol/mol).

### Oxidative stress

**Fig. 3:**
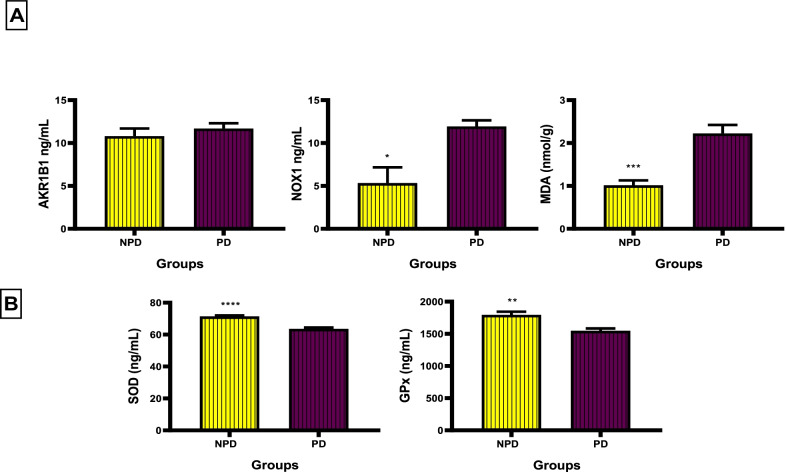
The concentration of AKR1B1, NOX1, MDA, SOD and GPx between the NPD and PD group. Values are presented as mean ± SEM. (n = 6 in each group).** A** NOX1: * Indicates *p* = 0.0156. MDA *** Indicates *p* = 0.0007. GPx ** *p* = 0.0033.** B** SOD **** indicates *p* < 0.0001. NPD, non-prediabetes; PD, prediabetes; AKR1B1, aldose reductase; NOX1(NADH oxidase 1), nicotinamide adenine dinucleotide phosphate oxidase 1; MDA, malondialdehyde; SOD, superoxide dismutase; GPx, glutathione peroxidase

The concentration of AKR1B1, NOX1, MDA, SOD and GPx was measured between the NDP and PD group at the end of the prediabetes induction period. Figure 3 depicts an increase in oxidative stress as indicated by an increase in oxidative stress biomarkers and a decrease in antioxidant enzymes. Figure 3A shows an insignificant increase in the concentration of AKR1B1 (*p* = 0.4419) and a significant increase in NOX1 (*p* = 0.0156) and MDA (*p* = 0.0007) concentration in the PD group compared to the NPD group**.** Figure 3B shows a significant decrease in the concentration of SOD (SOD_NPD_ = 71.44 ± 0.59 ng/mL and SOD_PD_ = 63.61 ± 0.83 ng/mL) and GPx (GPx_NPD_ = 1791 ± 47.04 ng/mL and GPx_PD_ = 1547 ± 37.89 ng/mL) in the PD group compared to the NPD group with *p* < 0.0001 and *p* = 0.0033 respectively.

### Cardiac injury

#### Cardiac troponins (cTnT and cTnI)

**Fig. 4:**
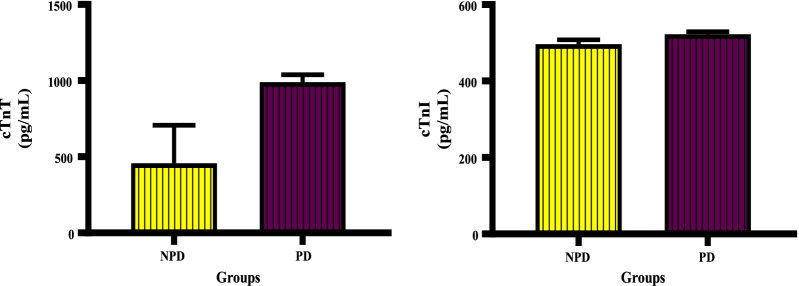
The concentration of cardiac troponins between the NPD and PD group. Values are presented as mean ± SEM. (n = 6 in each group). NPD, non-prediabetes; PD, prediabetes; cTnT, cardiac troponin T; cTnI, cardiac troponin I

The concentration of cTnT and cTnI was measured between the NPD and PD groups at the end of the prediabetes induction period. Figure 4 shows that there was an increase in mean cTnT concentration (989.5 ± 48.60 pg/mL) in the PD group compared to NPD group (456.50 ± 280.10 pg/mL); however, the increase was insignificant (*p* = 0.0814). The concentration of cTnI was increased (522.20 ± 6.30 pg/ml) in the PD group compared to the NDP group (496.20 ± 11.34 pg/mL). The increase insignificant (*p* = 0.0799).

#### Myocardial morphology

**Fig. 5:**
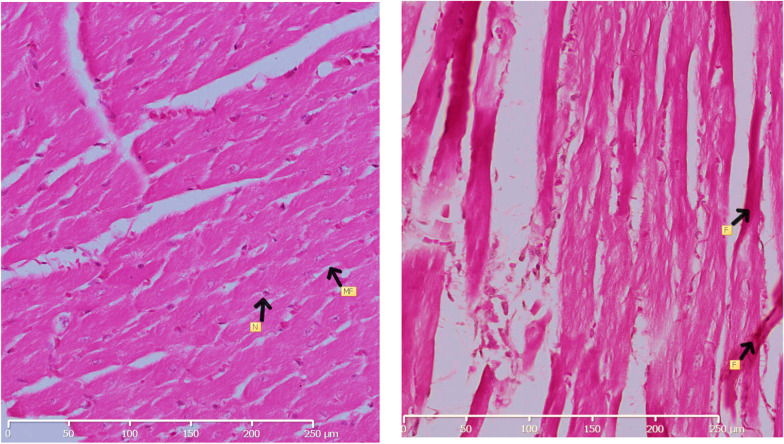
Illustrates H & E photomicrographs from the myocardium of the NPD and PD group. NPD, non-prediabetes; PD, prediabetes; MF, myofiber; N, nucleus; F, fibrous fibres

Heart tissue was processed for histological analysis at the end of the prediabetes induction period. Figure 5 shows H & E photomicrographs from the NPD (A) and PD (B) myocardium at a 20X250µm magnification. In Fig. 5A, the myocardium has a regular shape of myofibers and a nucleus that is located centrally. In Fig. 5B, myofibers are disarrayed and consist of fibrous fibres.

#### Correlation

The association between HbA1c and the cardiac troponins (cTnT and cTnI) was calculated between the NPD and PD group. There was an insignificant negative association between HbA1c and cTnT as well as cTnI in the NPD group (r = − 0.74; *p* = 0.2552) and (r = − 0.84; *p* = 0.0722) respectively. The association between HbA1c and cTnT including cTnI was positive and insignificant in the PD group (r = 0.37; *p* = 0.6273) and (r = 0.06; *p* = 0.9188) respectively.

## Discussion

T2DM is a significant risk factor for CVD [34, 35]. However, T2DM is preceded by prediabetes which is an intermediate state of hyperglycaemia [3]. Complications associated with T2DM are reported to begin during the prediabetes stage [36, 37]. Studies in our laboratory have demonstrated that hepatic and renal dysfunction and injury seen in T2DM are present during prediabetes [38, 39]. However, the risk factors of myocardial tissue injury have not been investigated in diet-induced prediabetes. Hence this study was conducted to assess myocardial tissue injury in diet-induced prediabetes.

Hyperglycaemia is due to impaired insulin secretion or impaired insulin because of pancreatic beta-cell dysfunction or insulin resistance [40]. In states of insulin resistance, beta-cells compensate by increasing insulin secretion to maintain glucose homeostasis. However, insulin levels increase and glucose remains elevated [41]. Insulin resistance and hyperinsulinemia result in impaired glucose tolerance [42]. IFG is obtained from fasting plasma glucose and occurs because of poor glucose regulation, resulting in raised plasma glucose even after an overnight fast. [42]. IGT is associated with peripheral insulin resistance, and IFG is associated with hepatic insulin resistance and endogenous glucose production [4]. According to a study by Bacha and colleagues, obese adolescents who show signs of glucose dysregulation, including IFG, IGT, or both, are more likely to have impaired insulin secretion rather than reduced insulin sensitivity [3]. HbA1c is a product of a non-enzymatic reaction between glucose and haemoglobin. HbA1c increases as the average plasma glucose level increase and reflects average plasma glucose over a long period [43, 44]. In the present study, the PD group depicted a significantly impaired glucose tolerance, FBG and HbA1c concentration compared to the NPD group. These results coincide with the results published by Siboto et al., [45]. The PD group also had a significantly higher body weight and heart weight compared to the NPD. High body weight and heart weight can be attributed to the high calorie consumed for 20 weeks.

Aldose reductase (AR) reduces glucose to fructose in the polyol pathway, thus shifting excess glucose from metabolism [46]. This reaction reduces NADPH which is essential for glutathione formation while producing NADH, a substrate for NADH oxidase [12]. Activation of AR puts a strain on the NADPH system and subsequently on the glutathione reductase/glutathione peroxidase system, which protects against oxidative stress [47, 48]. In this study, there was an insignificant increase in AKR1B1 concentration in PD compared to the NDP group. These results contradict the finding by Daniels et al., who recently reported a significant increase in cardiac fructose and sorbitol in T2DM subjects. Sorbitol positively correlated with diastolic dysfunction. In Zucker Diabetes Fatty rats, fructose metabolism enzymes were markedly increased [49]. This study analysed intermediates of the polyol and then inferred that the enzymes of the polyol pathway are increased since intermediates of the pathway are elevated. AR reductase is active in chronic hyperglycaemia. The present study analysed AR in intermediate hyperglycaemia. Intermediate hyperglycaemia could be the reason that we did not find evidence of statistical significance.

NOX 1 is one of the cellular sources of ROS [50]. NOX1 catalyses the transfer of electrons to O_2_ generation O^2−^ (superoxide) and H_2_O_2_ (hydrogen peroxide) [51]. Hyperglycaemia activates NOXI through PKC-dependent Rac1 activation [52]. NOX1 is also activated by the activation of the polyol pathway [12, 53]. In this study, there was a significant increase in NOX1 concentration in PD compared to the NPD group. These results coincide with the findings by Xu et al., in which wild type mice fed high-fat-high sucrose (HFHS) diet demonstrated an increase in Mac-2, IL-1β and nitrosative stress in cardiac tissue by comparison to NOX-1 knockout mice, indicating that NOX1 contributes to oxidative stress, endothelial activation and myocardial inflammation [54]. According to literature, ROS generation results in cardiac oxidative stress, associated with increased cardiac fibrosis, reduced cardiac contractility and ultimately cardiac dysfunction [55]. NOX1-induced ROS plays a role in endotoxin-induced cardiomyocyte apoptosis [22]. NOX1 elevation risks myocardial injury and myocardial infarction by inducing oxidative stress and inflammation.

Lipids present in plasma and cell membranes are subjected to ROS attack and peroxidation. Lipid peroxidation products are toxic to a cell and require removal by GSH [53]. MDA is a biomarker of oxidative stress formed as a lipid peroxidation product [56]. The level of MDA is increased in T2DM [57]. In the present study, there was a significant increase in MDA concentration in PD compared to the NPD group. These results are in accordance with the results reported by Su et al., in which subjects with diabetes and those with prediabetes had a significantly increased MDA concentration and a decreased SOD activity compared to subjects with standard glucose tolerance [58]. In the HFHC diet-induced prediabetes model, Akinnuga et al., reported a significant increase in MDA concentration in the prediabetes group compared to non-prediabetes [30].

Reactive oxygen species (ROS) are highly reactive molecules that regulate vascular tone and cell proliferation [59]. Antioxidant enzymes prevent abnormal ROS production and lipid peroxidation. Enzymes such as SOD converts superoxide to hydrogen peroxide, which is then transformed into water by catalase or glutathione peroxidase (GPx) [60]. Glutathione reductase is a hydrogen donor to GPx; therefore, it is vital for the activity of GPx [61]. In this study, there was a significant decrease in SOD and GPx. These results concur with the results published by Mabuza et al., in which the prediabetes group showed a significant decrease in SOD and GPx in the cardiac tissue [33]. An increase in AR activity is one of the many factors that cause a decrease in GPx. AR depletes NADH, a substrate for GSH. AR metabolizes GSH-lipid-derived aldehyde adducts, which decreases GSH and subsequently increases oxidative stress [61].

The myocardium releases cardiac troponins (cTn) in proportion to the degree of myocardial tissue injury and disruption of the myocyte membrane [62–64]. Though these cardiac injury markers are usually tested in the event of cardiac ischemia, several studies have reported an elevation of these markers in subclinical myocardial damage in diabetes [37, 65]. A study by Selvin et al., demonstrated an incidence of elevated cTn in diabetic people [66]. Elevated cTn has a strong correlation with the adverse cardiovascular outcome whether a coronary disease is present or not [67]. In Table 1, there was an insignificant increase in cTnT and cTnI concentration between the PD and NPD groups in the current study. Interestingly, the concentration of cTnT in PD (989.5 ± 48.6 pg/mL) was double the concentration in NPD (456.5 ± 250.1 pg/mL) whereas the concentration of cTnI in PD (522.2 ± 6.3 pg/mL) is 1 × factor higher than the concentration in NPD (496.2 ± 11.3 pg/mL). These results suggest cTnT is markedly elevated and is a sensitive marker of cardiac injury in prediabetes. In Table 1, we reported an insignificant negative association between HbA1c and the cardiac troponin (cTnT and cTnI) in the NPD group, whereas the association was positive in the PD group. This study is per a recently published study by Witkowski et al., in which prediabetes is associated with major adverse cardiac events (MACE) and increasing hs-cTnT levels associated with an increased risk for 3-year MACE and 5-year all-cause mortality in the entire cohort [68].Interestingly, there was no correlation between the levels of hs-cTnT and either FBG or HbA1c [69]. Matsumoto observed a significant positive correlation between HbA1c and hs-cTnT in participants with T2DM. The concentration of hs-cTnT was further positively associated with oxidative stress markers [70]. These findings contradict the findings of this study. We suggest that this could be the difference in the study groups, methodology, and hyperglycaemia duration.

**Table 1:** Correlation between antioxidant enzymes and oxidative stress biomarkers

	cTnT (pg/mL)		cTnI (pg/mL)	
	NPD	PD	NPD	PD
HbA1c	− 0.74	0.37	− 0.84	0.06
*p* value	0.2552	0.6273	0.0722	0.9188

Values are represented as Pearson r value. (n = 6 in each group).

*NPD* non-prediabetes, *PD* prediabetes, *HbA1c* glycated haemoglobin, *cTnT* troponin T, *cTnI* troponin I

In a cross-sectional study conducted by Kerr and colleagues, there was a significant increase in cTnI levels in T2DM compared to non-diabetes subjects [71]. These results are different from the findings of this study reported in Table 1. We did not find evidence of a statistical difference in the concentration of cTnI between the NPD and the PD group. We speculate this to be because the study consisted of 6 rats per group and had intermediate hyperglycaemia whereas Kerr reported a cross-sectional study with T2DM participants. Odum and Young investigated the level of cTnI, CK-MB and myoglobin and their relation to CVD risk in T2DM. None of the participants had an elevation of all three biomarkers, and only 4.3% had an elevation of two biomarkers [72]. In the present study, we analysed cTnT and cTnI. Though there was an insignificant increase, the concentration of cTnT in the PD group doubled the concentration in the NPD group, whereas the concentration of cTnI increased by 1. This difference in cTn concentration suggests that testing one biomarker may not detect the risk or detect the injury.

In a study associating subclinical myocardial injury with arterial stiffness in T2DM patients, hs-cTnI had an insignificant positive correlation with HbA1c and arterial stiffness [73]. These studies follow the present study as we did not find a statistically significant association between HbA1c and cTnI. Detection of cardiac biomarkers in prediabetes indicates a subclinical myocardial injury during prediabetes.

Elevated ROS in the cardiac tissue results in cardiac oxidative stress, cardiac fibrosis, cardiac dysfunction and potentially cardiac events such as MI [55]. This study observed increased AR, NOX1, MDA, and decreased antioxidant enzymes in the PD group compared to the NPD group myocardium. Oxidative stress through the polyol pathway is reported to decrease SERCA activity, thereby decreasing Ca^2+^and subsequently resulting in contractile dysfunction [74]. Contractile dysfunction is also caused by myocardial apoptosis and fibrosis [75]. Studies have demonstrated that myocardial fibrosis and deranged myofibers are hyperglycaemia induced myocardial structural changes [76]. In the present study, in Fig. 5 we observed disarrayed myofibers and the deposition of fibrous networks in the PD myocardium. In contrast, the NPD group's nucleus is centrally located, and the myofibers are regularly shaped. This observation aligns with Kusaka et al., who reported that the cardiac tissue of the SHrcp rat model of metabolic syndrome with prediabetes displayed oxidative stress, inflammation, hypertrophy, and fibrosis compared to the control [77].

## Conclusion

The findings of this study demonstrate that prediabetes is associated with myocardial injury through oxidative stress. Future studies are to use immunohistochemistry to investigate the cardiac contractile function and include more cardiac biomarkers.

## Supplementary Information

**Additional file 1**. The association between antioxidant enzymes (SOD and GPx) and oxidative stress biomarkers (NOX1 and MDA) was calculated between the NPD and PD group. In the NPD group there was an insignificant positive correlation (SOD and NOX1: r = 0.47,* p* = 0.5268), (SOD and MDA: r = 0.72,* p* = 0.1741), (GPx and NOX1: r = 0.70,* p* = 0.3033), (GPx and MDA: 0.23,* p* = 0.6908) whereas in the PD group there was an insignificant negative association (SOD and NOX1: r = −0.72,* p* = 0.2846), (SOD and MDA: r = −0.66,* p* = 0.2218), (GPx and NOX1: r = −0.91,* p* = 0.0950), (GPx and MDA: r = −0.22,* p* = 0.7240) between the antioxidant enzymes and oxidative stress biomarkers.

## Data Availability

The datasets generated during and analysed during the current study are not publicly available due to concerns of misuse and the more aspects of the study are to be added. However, datasets are available from the corresponding author on reasonable request.

## Abbreviations

%: Percentage
ADA: American Diabetes Association
AGE: Advanced glycation end-product
AKR1B1: Aldo-keto reductase family 1, member 1
ANGΙΙ: Angiotensin 2
AR: Aldose reductase
AREC: Animal Research Ethics Committee
AUC: Area under the curve
BRU: Biomedical Research Unit
Ca^2+^: Calcium
CAT: Catalase
CK-MB: Creatine kinase-myocardial band
Co.: Company
cTn: Cardiac troponin
cTnI: Cardiac troponin I
cTnT: Cardiac troponin T
CVD: Cardiovascular disease
ELISA: Enzyme-linked immunosorbent assay
eNOS: Endothelial nitric oxide synthase
FBG: Fasting blood glucose
GPx: Glutathione reductase
GSH: Glutathione
H&E: Haematoxylin and eosin
H_2_O_2_: Hydrogen peroxide
HbA1c: Glycated haemoglobin
HFD: High-fat diet
HFHC: High-fat-high-carbohydrate
HFHS: High-fat-high-sucrose
HNE: 4-Hydroxynonenal
Hr: Hour
hs-cTnT: High sensitivity cardiac troponin T
IFG: Impaired fasting glucose
IGT: Impaired glucose tolerance
IL-1β: Interleukin 1-beta
Kg: Kilogram
Ltd: Limited
MACE: Major adverse cardiac event
MDA: Malondialdehyde
MI: Myocardial infarction
min: Minute
mmol/L: Millimoles per litre
mRNA: Messenger ribonucleic acid
NADH: Reduced nicotinamide adenine dinucleotide
NOX1: NADPH oxidase 1 encoding gene
NPD: Non-prediabetes
O_2_: Oxygen
O^2^^−^: Superoxide ion
OGTT: Oral glucose tolerance test
pg: Picogram
PKC: Protein kinase C
r: Pearson’s correlation coefficient
Rac1: Ras-related C3 botulinum toxin substrate 1
ROS: Reactive oxygen species
SDH: Sorbitol dehydrogenase
SEM: Standard error of the mean
SERCA: Sarco/endoplasmic reticulum Ca^2+^-ATPase
SOD: Superoxide dismutase
T2DM: Type 2 diabetes mellitus
TX: Texas
UKZN: University of KwaZulu Natal
USA: United States of America
