# Supplementary material for: Assessing the risk factors for myocardial infarction in diet-induced prediabetes: myocardial tissue changes
Source: BMC Cardiovasc Disord. 2022 Aug 2;22:350. doi: 10.1186/s12872-022-02758-8 (PMC9347129; doi:10.1186/s12872-022-02758-8)
Supplement: Supplementary file 1 — Additional file 1. The association between antioxidant enzymes (SOD and GPx) and oxidative stress biomarkers (NOX1 and MDA) was calculated between the NPD and PD group. In the NPD group there was an insignificant positive correlation (SOD and NOX1: r = 0.47, p = 0.5268), (SOD and MDA: r = 0.72, p = 0.1741), (GPx and NOX1: r = 0.70, p = 0.3033), (GPx and MDA: 0.23, p = 0.6908) whereas in the PD group there was an insignificant negative association (SOD and NOX1: r = −0.72, p = 0.2846), (SOD and MDA: r = −0.66, p = 0.2218), (GPx and NOX1: r = −0.91, p = 0.0950), (GPx and MDA: r = −0.22, p = 0.7240) between the antioxidant enzymes and oxidative stress biomarkers. [file 12872_2022_2758_MOESM1_ESM.pdf]

Table 1: Correlation between oxidative stress biomarkers and antioxidant enzymes

	SOD		GPx	
	NPD	PD	NPD	PD
NOX1	0.47	-0.72	0.70	-0.91
p value	0.5268	0.2846	0.3033	0.0950
MDA	0.72	-0.66	0.23	-0.22
p value	0.1741	0.2218	0.6908	0.7240

Values are represented as Pearson r value. (n = 6 in each group). NPD, non-prediabetes; PD, prediabetes; SOD, superoxide dismutase; GPx, glutathione peroxidase; NOX1(NADH oxidase 1), nicotinamide adenine dinucleotide phosphate oxidase 1; MDA, malondialdehyde
